# From Gram-Negative Neonatal Sepsis to Neurodevelopmental Impairment: A Retrospective Cohort Study in Preterm Infants

**DOI:** 10.3390/children13070850

**Published:** 2026-06-24

**Authors:** Mihaela Zaharie, Marioara Boia, Aniko Manea, Roxana Maria Jeleriu, Mirabela Adina Dima, Ileana Enatescu, Daniela Iacob

**Affiliations:** 1 Doctoral School, “Victor Babes” University of Medicine and Pharmacy Timisoara, 300041 Timisoara, Romania; mihaela.zaharie@umft.ro (M.Z.); roxana.jeleriu@umft.ro (R.M.J.); 2 Department of Neonatology and Puericulture, “Victor Babes” University of Medicine and Pharmacy Timisoara, 300041 Timisoara, Romania; manea.aniko@umft.ro (A.M.); dima.mirabela@umft.ro (M.A.D.); enatescu.ileana@umft.ro (I.E.); iacob.daniela@umft.ro (D.I.); 3 Neonatology and Preterm Department, “Louis Ţurcanu” Children Emergency Hospital, 300011 Timisoara, Romania; 4 Clinic of Neonatology, “Pius Brinzeu” County Clinical Emergency Hospital, 300723 Timișoara, Romania; 5 Center for Multidisciplinary Research and Management in Pediatric Surgical Conditions (CCMMA–CHIR-PED), 300011 Timisoara, Romania

**Keywords:** Gram-negative sepsis, preterm infants, neonatal infection, neurodevelopmental outcomes, Bayley scales, multidrug-resistant bacteria, biomarkers, CRP, procalcitonin, neonatal intensive care

## Abstract

**Highlights:**

**What are the main findings?**

- Within this cohort of culture-proven Gram-negative neonatal sepsis, late-onset infections predominated, and *Klebsiella pneumoniae* was the leading pathogen.
- Multidrug-resistant organisms were associated with 52.0% of infections, and in-hospital mortality reached 26.0%.

**What are the implications of the main findings?**

- Neurodevelopmental impairment was associated with 38% of survivors at 18–24 months’ corrected age, predominantly affecting language and cognitive domains.
- Lower gestational age emerged as the strongest independent predictor of both mortality and neurodevelopmental impairment, supporting early recognition, targeted antimicrobial therapy, and structured long-term follow-up.

**Abstract:**

Background/Objectives: Gram-negative neonatal sepsis remains a cause of morbidity and mortality in preterm infants, yet the relationship between early clinical severity and long-term neurodevelopmental outcomes is incompletely defined. This study aimed to characterize Gram-negative sepsis in preterm infants and to evaluate its short-term and 18–24-month neurodevelopmental consequences. Methods: We conducted a retrospective observational cohort study of preterm infants admitted to a tertiary neonatal intensive care unit between 1 January 2022 and 31 December 2023. Infants with culture-proven Gram-negative neonatal sepsis, including both early-onset sepsis (EOS) and late-onset sepsis (LOS), were included. Clinical, microbiological, therapeutic, and laboratory data were collected, and survivors were assessed at 18–24 months’ corrected age using the Bayley Scales of Infant and Toddler Development. Results: Among infants with culture-proven Gram-negative sepsis, late-onset cases were more frequent than early-onset cases, and *Klebsiella pneumoniae* was the most common pathogen (38.0%). Multidrug-resistant organisms were associated with 52.0% of infections. In-hospital mortality was 26.0%. Major short-term complications included intraventricular hemorrhage (24.0%), severe intraventricular hemorrhage (20.0%), necrotizing enterocolitis (12.0%), bronchopulmonary dysplasia (20.0%), and meningitis (10.0%). Among survivors who underwent neurodevelopmental assessment, neurodevelopmental impairment was observed in 38.0%, most frequently affecting the language (22.5%) and cognitive (20.0%) domains. Infants with neurodevelopmental impairment had significantly lower gestational age and birth weight and higher inflammatory biomarker levels. In multivariable analyses, lower gestational age emerged as the strongest independent predictor of both mortality (adjusted OR 0.19, 95% CI 0.04–0.99) and neurodevelopmental impairment (adjusted OR 0.12, 95% CI 0.02–0.71). Conclusions: Gram-negative neonatal sepsis in preterm infants was associated with substantial mortality, severe neonatal complications, and a high burden of later neurodevelopmental impairment. Lower gestational age was independently associated with adverse short- and long-term outcomes. These findings support early recognition, targeted antimicrobial therapy, and structured neurodevelopmental follow-up in this high-risk population.

## 1. Introduction

Preterm birth, defined as delivery before 37 weeks’ gestation, affected an estimated 13.4 million infants worldwide in 2020 and remains the leading cause of death among children under five years of age, accounting for approximately 900,000 deaths annually worldwide [1,2,3]. Preterm infants exhibit heightened vulnerability to infections due to immature innate and adaptive immunity, underdeveloped skin barriers, and frequent invasive interventions, elevating risks of early- and late-onset sepsis (EOS, LOS). Neonatal sepsis carries profound clinical significance worldwide, with reported mortality rates generally ranging from 11% to 19%, although substantial variation exists across geographic regions, healthcare systems, and neonatal intensive care unit (NICU) settings [4].

Neonatal sepsis is associated with both acute and long-term complications. Short-term consequences include respiratory failure, necrotizing enterocolitis, hemodynamic instability, intraventricular hemorrhage (IVH), and bronchopulmonary dysplasia (BPD), whereas long-term sequelae include neurodevelopmental impairment, cognitive deficits, and chronic health problems [5,6].

Gram-negative bacteria are a major cause of sepsis in preterm infants, with *Escherichia coli* and *Klebsiella* spp. as the most common pathogens. In many NICU cohorts, particularly those from low- and middle-income countries, Gram-negative infections are associated with higher mortality rates (up to 25%), increased need for respiratory support, and higher inflammatory biomarker levels, including C-reactive protein (CRP) and procalcitonin (PCT) [7,8,9,10].

Antimicrobial resistance patterns vary considerably across regions; however, several studies from low- and middle-income settings have reported resistance rates exceeding 90% for ampicillin–gentamicin and 40–70% for third-generation cephalosporins [11,12].

Inflammatory biomarkers such as CRP and PCT are widely used to support the diagnosis and monitoring of neonatal sepsis. Beyond their diagnostic role, these biomarkers may also provide prognostic information regarding disease severity and adverse outcomes [13,14,15,16].

Neonatal sepsis in preterm infants precipitates severe acute complications, including intraventricular hemorrhage (IVH), necrotizing enterocolitis (NEC), and bronchopulmonary dysplasia (BPD), with mortality risks escalating inversely with gestational age—reaching 30–35% in very low birth weight cases [17]. Nearly 80% of sepsis-attributable deaths involve concurrent severe IVH grade 3/4, periventricular leukomalacia, or NEC, often prompting comfort care transitions [18].

Long-term neurodevelopmental impairment has been reported in up to 38% of survivors in selected cohorts of preterm infants with neonatal sepsis, although estimates vary substantially depending on population characteristics, follow-up duration, and assessment methods [19,20]. Neurodevelopmental assessment is commonly performed using the Bayley Scales of Infant and Toddler Development (BSID), which evaluate cognitive, language, and motor domains and provide standardized measures of developmental performance at 18–24 months’ corrected age [21,22].

Despite advances in neonatal care, important knowledge gaps remain. Most studies evaluate neonatal sepsis as a heterogeneous entity, whereas relatively few focus specifically on Gram-negative infections and their distinct microbiological characteristics, antimicrobial resistance patterns, and clinical outcomes. Furthermore, studies that simultaneously integrate microbiological findings, inflammatory biomarkers, short-term complications, and standardized long-term neurodevelopmental assessments remain limited. Consequently, the specific contribution of Gram-negative pathogens to both acute morbidity and later neurodevelopmental impairment in preterm infants remains incompletely understood.

The novelty of the present study lies in its comprehensive evaluation of culture-proven Gram-negative neonatal sepsis in a cohort of preterm infants, including both early-onset and late-onset disease. By combining clinical, microbiological, antimicrobial resistance, biomarker, short-term outcome, and 18–24-month Bayley neurodevelopmental data within a single analytical framework, this study provides an integrated assessment of factors associated with adverse outcomes in this high-risk population.

Therefore, the present retrospective cohort study aimed to characterize Gram-negative neonatal sepsis in preterm infants, including both early-onset (EOS) and late-onset (LOS) disease. By integrating clinical, microbiological, and biomarker data with short-term complications and neurodevelopmental outcomes assessed at 18–24 months using the Bayley Scales, we sought to provide a comprehensive evaluation of this high-risk population and identify factors associated with adverse outcomes.

## 2. Materials and Methods

### 2.1. Study Design

This retrospective observational cohort study adhered to the STROBE reporting guidelines. Preterm infants (<37 weeks’ gestation) admitted to the tertiary neonatal intensive care unit (NICU) of “Louis Turcanu” Children’s Hospital, Timisoara, between 1 January 2022 and 31 December 2023 with blood culture-proven Gram-negative neonatal sepsis (EOS and LOS) were included. Cases were identified via electronic health records using ICD-10 codes (P36.0–P36.9) and microbiology laboratory databases. Exclusion criteria encompassed major congenital anomalies and transfer after 48 h of life.

Clinical, microbiological, therapeutic, and short-term outcome data were extracted from medical charts. Neurodevelopmental assessments using the Bayley Scales of Infant and Toddler Development (BSID-III/IV) were performed at 18–24 months’ corrected age, with composite scores for cognitive, language, and motor domains. Follow-up attendance was tracked via clinic records. Data analysis aimed to evaluate infection characteristics, antimicrobial management, acute complications, and long-term neurodevelopmental outcomes. The study received approval from the Research Ethics Committee of Victor Babeș University of Medicine and Pharmacy, Timișoara, Romania (Approval No. 107/6 July 2020, revised in 2025), and from the Research Ethics Committee of “Louis Turcanu” Children’s Hospital, Timișoara (Approval No. 133/2025).

### 2.2. Study Population

Preterm infants were defined as those born before 37 weeks’ gestational age, consistent with standard neonatal classifications.

**Figure 1:**
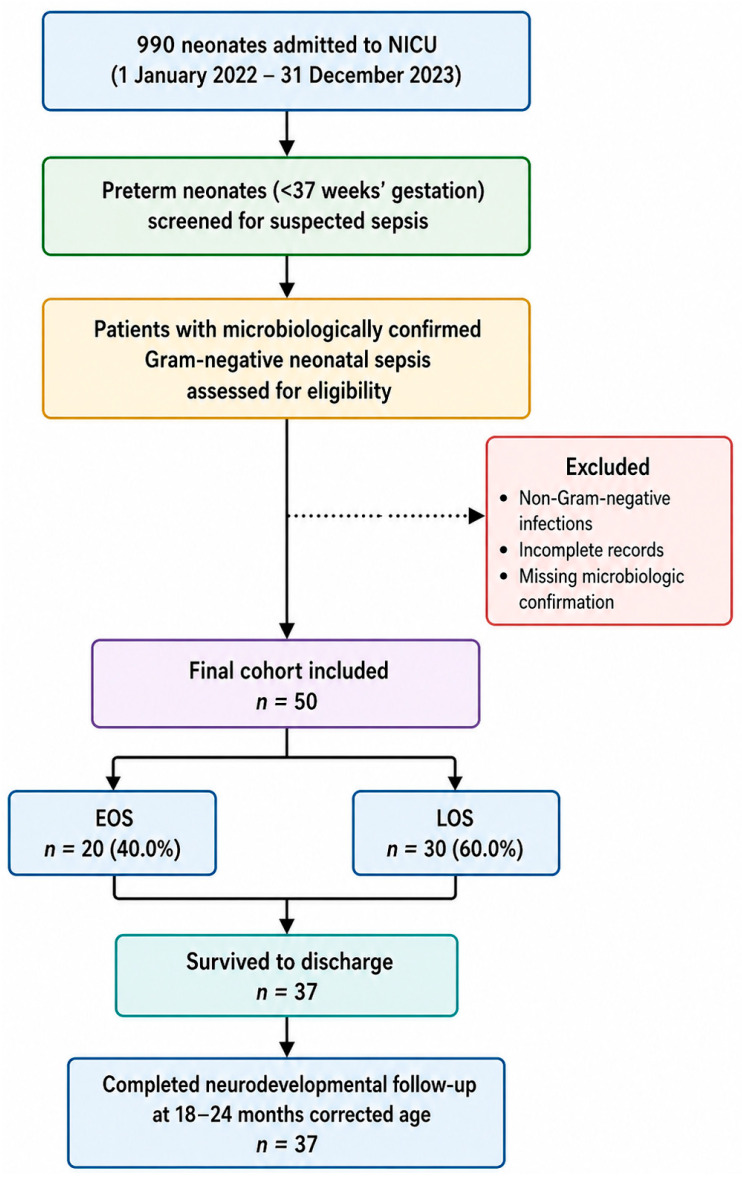
Flowchart illustrating cohort identification, the inclusion and exclusion process, survival status, and availability of neurodevelopmental follow-up.

Of 990 newborns admitted to the NICU during the study period, 50 (5.1%) met the inclusion criteria for culture-proven Gram-negative neonatal sepsis (including both EOS and LOS) in preterm infants after applying exclusion criteria, forming the final cohort (Figure 1).

Inclusion criteria required NICU admission with clinical signs of sepsis—including temperature instability, poor feeding, tachypnea, respiratory distress, lethargy, hypotension, or apnea—and microbiological confirmation of Gram-negative neonatal sepsis, defined as symptom onset within 72 h of life and LOS after 72 h. Confirmation necessitated positive cultures from blood, cerebrospinal fluid (CSF), or urine, identifying Gram-negative pathogens such as *Escherichia coli*, *Klebsiella pneumoniae*, *Pseudomonas aeruginosa*, *Proteus mirabilis*, *Serratia marcescens*, *Acinetobacter baumannii*, *Enterobacter cloacae*, *Citrobacter freundii*, or *Morganella morganii*, as documented in laboratory reports.

Exclusion criteria included non-Gram-negative infections, such as Gram-positive bacterial or fungal infections, polymicrobial infections without a predominant Gram-negative etiology, and major congenital anomalies. Cases with incomplete microbiological data or lacking culture confirmation were excluded to ensure diagnostic rigor.

### 2.3. Definitions

EOS was defined as culture-proven Gram-negative bloodstream infection with symptom onset within the first 72 h of life, whereas LOS was defined as infection occurring after 72 h of life. Only cases with microbiological confirmation from blood cultures were included.

Multidrug-resistant (MDR) organisms were defined as Gram-negative bacteria exhibiting resistance to at least one agent in three or more antimicrobial classes, according to internationally accepted criteria.

Major neonatal morbidities were defined according to established clinical and radiological criteria. Intraventricular hemorrhage (IVH) was diagnosed by cranial ultrasonography and graded according to the Papile classification, with severe IVH defined as grade III or IV. Necrotizing enterocolitis (NEC) was diagnosed based on modified Bell staging criteria, and only stage II or higher was considered clinically significant. Bronchopulmonary dysplasia (BPD) was defined as oxygen dependency at 36 weeks’ postmenstrual age. Meningitis was diagnosed based on compatible clinical findings and cerebrospinal fluid analysis and/or positive microbiological culture results.

Neurodevelopmental impairment (NDI) was assessed at 18–24 months’ corrected age using the Bayley Scales of Infant and Toddler Development, third or fourth edition (BSID-III/BSID-IV). NDI was defined as the presence of at least one of the following: cognitive, language, or motor composite score below 85; cerebral palsy; severe visual impairment; or hearing impairment requiring amplification. Corrected age was used for all neurodevelopmental assessments.

Mortality was defined as all-cause death occurring during initial hospitalization. Length of hospital stay was calculated as the number of days from admission to discharge or death.

### 2.4. Data Collection

Data were systematically extracted from electronic health records, microbiology laboratory databases, and neurodevelopmental follow-up clinic files by two independent investigators, with discrepancies resolved through consensus.

Maternal and perinatal variables included maternal age, multiple gestation, prolonged rupture of membranes (>18 h), maternal fever, clinical chorioamnionitis, antenatal antibiotic exposure, and antenatal corticosteroid administration.

Neonatal baseline characteristics comprised gestational age, birth weight, sex, mode of delivery, Apgar scores at 1 and 5 min, age at sepsis onset, and clinical manifestations at presentation.

Laboratory data collected at sepsis diagnosis included CRP, PCT, white blood cell count, and platelet count.

Microbiological data included culture source, identified Gram-negative pathogens, antimicrobial susceptibility testing results, and MDR status.

Therapeutic variables included empirical and targeted antimicrobial therapy, aminoglycoside and carbapenem exposure, duration of antibiotic treatment, central venous catheter placement, mechanical ventilation, continuous positive airway pressure (CPAP) support, inotropic therapy, and parenteral nutrition.

Clinical outcome data included major neonatal morbidities, duration of hospitalization, weight at discharge, corrected gestational age at discharge, hospital readmission within 6 months, and in-hospital mortality.

All study data were entered into a secure Excel database and independently verified through double data checking before analysis.

### 2.5. Microbiological Assessment

Microbiological evaluation was performed as part of routine clinical care in all infants with suspected neonatal sepsis. Blood cultures were obtained before initiation of antibiotic therapy whenever clinically feasible. Additional specimens, including cerebrospinal fluid and urine cultures, were collected when indicated by the clinical presentation and attending physician.

Gram-negative pathogens were identified using standard microbiological laboratory procedures employed by the hospital microbiology department. Antimicrobial susceptibility testing was performed in accordance with contemporary laboratory standards, and susceptibility profiles were recorded for all culture-confirmed isolates.

MDR organisms were defined as Gram-negative bacteria exhibiting resistance to at least one antimicrobial agent in three or more antibiotic classes.

Antimicrobial management followed local NICU protocols and clinical judgment. Empirical antibiotic therapy was initiated immediately after clinical suspicion of sepsis and collection of microbiological samples. Initial empirical therapy generally consisted of ampicillin plus gentamicin for suspected neonatal sepsis, whereas broader-spectrum regimens, including meropenem-based combinations, were considered for critically ill infants or those at increased risk of multidrug-resistant Gram-negative infection.

Following microbiological identification and antimicrobial susceptibility testing, antibiotic regimens were reviewed and modified when appropriate. Escalation of therapy was considered in cases of clinical deterioration, persistent inflammatory response, or identification of resistant pathogens, whereas de-escalation was performed when culture and antibiogram results supported the use of narrower-spectrum agents. The duration of antimicrobial therapy was individualized based on the site of infection, microbiological findings, and clinical response.

### 2.6. Neurodevelopmental Assessment

Neurodevelopmental follow-up was performed at 18–24 months’ corrected age in all surviving infants. Developmental outcomes were assessed using the Bayley Scales of Infant and Toddler Development, either the third edition (BSID-III) or the fourth edition (BSID-IV), depending on the version available at the follow-up clinic at the time of evaluation. BSID-III was administered in 32 infants and BSID-IV in five infants.

All assessments were performed using corrected age and were conducted by clinicians experienced in neonatal neurodevelopmental follow-up. Because evaluations were performed as part of routine clinical care, assessors were not formally blinded to neonatal clinical characteristics and hospitalization data.

The Bayley Scales provide standardized composite scores (mean 100, standard deviation 15) across cognitive, language, and motor domains. Because BSID-III and BSID-IV assess comparable developmental domains and use standardized normative scoring systems, pooled analyses were considered acceptable for exploratory outcome assessment.

Neurodevelopmental impairment (NDI) was defined as the presence of at least one of the following: a cognitive, language, or motor composite score below 85; cerebral palsy; severe visual impairment; or hearing impairment requiring amplification. Follow-up attendance and assessment completion were verified through outpatient clinic records.

No neurodevelopmental outcome data were missing among hospital survivors. All 37 infants who survived to discharge completed the neurodevelopmental assessment at 18–24 months’ corrected age; therefore, no imputation procedures or sensitivity analyses for missing neurodevelopmental outcomes were required.

### 2.7. Outcome Measures

The primary outcomes of the study were in-hospital mortality and NDI assessed at 18–24 months’ corrected age.

Secondary outcomes included major neonatal morbidities associated with Gram-negative sepsis, including intraventricular hemorrhage (IVH), severe IVH (grade III/IV), necrotizing enterocolitis (NEC), bronchopulmonary dysplasia (BPD), meningitis, duration of hospitalization, corrected gestational age at discharge, weight at discharge, and hospital readmission within the first 6 months of life.

Additional exploratory outcomes included the distribution of Gram-negative pathogens, prevalence of multidrug-resistant (MDR) organisms, antimicrobial treatment patterns, and clinical factors associated with mortality and neurodevelopmental impairment in a retrospective perspective.

### 2.8. Statistical Analysis

Data analysis utilized Python (version 3.12.3) for descriptive statistics, visualization, and inferential modeling.

Descriptive statistics: Continuous variables reported as medians (IQR); categorical variables as frequencies (%). Normality assessed via the Shapiro–Wilk test. Comparative analyses: Mann–Whitney U, χ^2^/Fisher’s exact tests for group differences (survivors vs. non-survivors, MDR vs. non-MDR).

Outcomes: Primary outcome (neurodevelopmental impairment) rates with 95% CIs. Secondary outcomes were compared similarly.

Multivariable modeling: Logistic regression modeled factors associated with the primary outcome (NDI) and mortality, with gestational age, birth weight, MDR status, and initial CRP/procalcitonin as covariates. Odds ratios (ORs) with 95% CIs were reported; goodness-of-fit was assessed via the Hosmer–Lemeshow test and AUC-ROC. Collinearity was checked (VIF < 5).

Non-parametric methods and bootstrapping were prioritized due to *n* = 50 (including both EOS and LOS cases; 37 with follow-up). Significance: *p* < 0.05 (FDR-corrected applied to exploratory subgroup analyses involving multiple comparisons).

Given the limited number of outcome events, multivariable analyses were considered exploratory and hypothesis-generating rather than confirmatory. Because neurodevelopmental outcome data were available for all surviving infants, the planned worst-case sensitivity analysis for missing follow-up data was not applicable and was therefore not performed.

## 3. Results

### 3.1. Cohort Selection and Baseline Characteristics

Between 1 January 2022 and 31 December 2023, a total of 990 neonates were admitted to the NICU. Preterm neonates with suspected neonatal sepsis were screened for eligibility. Following microbiological confirmation and application of the predefined inclusion and exclusion criteria, 50 preterm infants with Gram-negative neonatal sepsis were included in the final retrospective cohort.

Among the included patients, both early-onset sepsis (EOS) and late-onset sepsis (LOS) cases were associated, with LOS accounting for the majority of infections. The study cohort consisted predominantly of very preterm and low-birth-weight neonates, reflecting the increased susceptibility of this population to severe infectious complications.

Overall, 37 infants survived to hospital discharge, while in-hospital mortality was recorded in 13 cases (26.0%). Neurodevelopmental follow-up at 18–24 months’ corrected age was completed in 37 surviving infants (74.0% of the total cohort). No surviving infant was lost to neurodevelopmental follow-up; therefore, outcome analyses were performed on all 37 survivors. Neurodevelopmental assessment was performed using the Bayley Scales of Infant and Toddler Development (BSID-III/IV), which evaluate the cognitive, language, and motor domains.

Baseline demographic, perinatal, and maternal characteristics of the study population are summarized in Table 1. The analyzed variables included gestational age, birth weight, sex distribution, Apgar scores, mode of delivery, type of neonatal sepsis, and maternal infectious risk factors. A study flowchart illustrating cohort selection, exclusions, survival status, and follow-up availability is presented in Figure 1.

### 3.2. Clinical and Microbiological Characteristics of Gram-Negative Sepsis

All included neonates had microbiologically confirmed Gram-negative sepsis, with positive cultures documented in all 50 cases. LOS was identified in 30 cases (60.0%), and EOS in 20 (40.0%). As expected, age at sepsis onset differed significantly between groups: 28.4 h in EOS versus 411.7 h in LOS (*p* < 0.001).

*Klebsiella pneumoniae* was the most frequently isolated pathogen, identified in 19 cases (38.0%), followed by *Escherichia coli* in eight cases (16.0%) and *Serratia marcescens* in five cases (10.0%). The distribution of pathogens did not differ significantly between EOS and LOS groups (*p* = 0.336). *E. coli* was proportionally more frequent among EOS cases, whereas *Klebsiella pneumoniae* predominated in LOS.

Multidrug-resistant organisms were identified in 26 cases (52.0%). MDR prevalence was numerically higher in LOS than EOS, but this difference was not statistically significant (56.7% vs. 45.0%, *p* = 0.565). Initial inflammatory biomarker levels were higher in LOS, with median CRP values of 61.0 mg/L versus 35.6 mg/L in EOS (*p* < 0.001) and median procalcitonin values of 12.7 mg/L versus 7.1 mg/L (*p* < 0.001). Leukocyte counts did not differ significantly between groups, whereas platelet counts were significantly lower in LOS (*p* = 0.016) (Table 2).

### 3.3. Therapeutic Interventions and Supportive Care

Empirical antibiotic therapy varied according to clinical severity, timing of sepsis onset, and local antimicrobial resistance patterns. The most frequently administered empirical regimen was meropenem plus amikacin, used in 30 cases (60.0%), followed by ampicillin plus gentamicin in 11 cases (22.0%) and piperacillin–tazobactam plus amikacin in nine cases (18.0%).

Following microbiological confirmation and antimicrobial susceptibility testing, targeted therapy was adjusted accordingly. Meropenem-based regimens represented the predominant targeted therapeutic strategy, particularly among multidrug-resistant (MDR) infections. Overall, carbapenems were administered in 33 patients (66.0%), while aminoglycosides were used in all cases as part of either empirical or targeted therapy.

Supportive intensive care measures reflected the severity of infection in this cohort of preterm neonates. Central venous catheters were required in 35 cases (70.0%), parenteral nutrition in 36 cases (72.0%), and invasive mechanical ventilation in 21 cases (42.0%). Continuous positive airway pressure (CPAP) support was used in 10 patients (20.0%), whereas inotropic support was required in 15 neonates (30.0%).

Comparative analyses demonstrated that MDR infections were associated with significantly more frequent carbapenem use compared with non-MDR infections (100.0% vs. 29.2%, *p* < 0.001). In addition, antibiotic treatment duration was significantly longer in MDR cases (median 16 vs. 13 days, *p* = 0.004). Although LOS cases showed a tendency toward prolonged therapeutic support and increased use of invasive procedures, the duration of antibiotic therapy did not differ significantly between LOS and EOS groups (*p* = 0.213) (Table 3).

### 3.4. Short-Term Clinical Outcomes

Short-term clinical outcomes are summarized in Table 4. Within the study cohort, in-hospital mortality was recorded in 13 of 50 neonates (26.0%). Meningitis occurred in five cases (10.0%), necrotizing enterocolitis (NEC) in six cases (12.0%), and bronchopulmonary dysplasia (BPD) in 10 cases (20.0%). Intraventricular hemorrhage (IVH) was present in 12 neonates (24.0%), with severe IVH, defined as grade III–IV, identified in 10 cases (20.0%). Median hospitalization duration was 35.5 days (IQR: 28.0–50.8). Readmission by 6 months was documented in eight infants (16.0%).

In comparative analyses, non-survivors had a markedly higher frequency of IVH compared with survivors (100.0% vs. 5.0%; *p* < 0.001). Severe IVH was also restricted to the non-survivor group (100.0% vs. 0.0%; *p* < 0.001). NEC was more frequent among non-survivors (40.0% vs. 5.0%; *p* = 0.011), while hospitalization duration was shorter among non-survivors, reflecting early in-hospital death rather than faster recovery. After FDR correction, the association between mortality and IVH/severe IVH remained statistically significant.

**Figure 2:**
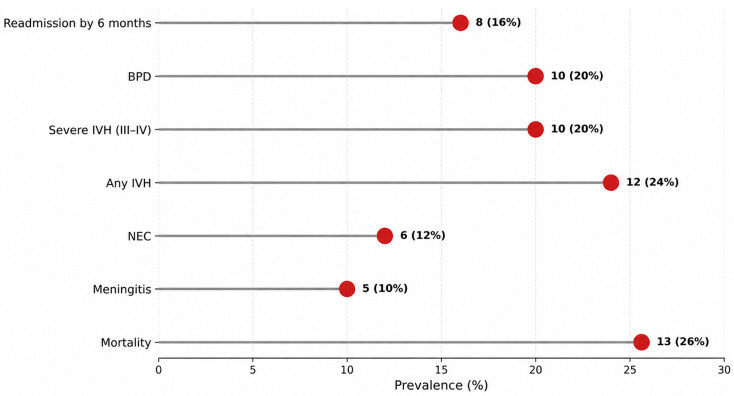
Distribution of major short-term complications and mortality among preterm infants with Gram-negative neonatal sepsis.

When outcomes were compared by MDR status, meningitis was observed only among MDR infections (19.2% vs. 0.0%), although this difference did not reach statistical significance after adjustment. Other short-term outcomes did not differ significantly between MDR and non-MDR cases. LOS cases showed a higher crude frequency of IVH and severe IVH than EOS cases, but these differences were not statistically significant after FDR correction (Table 5 and Figure 2).

Given the limited sample size, these findings should be interpreted cautiously.

### 3.5. Neurodevelopmental Outcomes at 18–24 Months

Neurodevelopmental follow-up data were available for all 37 infants who survived to discharge. The 13 infants who died during hospitalization were not eligible for the Bayley assessment and were excluded from the neurodevelopmental outcome analysis. Among survivors, the median corrected age at follow-up was 20.0 months (IQR: 18.0–22.0).

Bayley developmental assessments were performed using the BSID-III in 32 infants (86.5%) and the BSID-IV in five infants (13.5%). In the overall follow-up cohort, median composite scores were 97.0 (IQR: 86.0–101.0) for the cognitive domain, 92.0 (IQR: 86.0–96.0) for the language domain, and 95.5 (IQR: 86.0–99.3) for the motor domain.

Using the predefined threshold of any Bayley composite score <85, neurodevelopmental impairment was identified in 14 of 37 survivors (37.8%; 95% CI: 22–55). Severe impairment, defined as any composite score <70, was present in eight infants (20.0%; 95% CI: 10.5–34.8), while moderate impairment, defined as at least one composite score between 70 and 84 without any score <70, was observed in one infant (2.5%; 95% CI: 0.4–12.9).

Among affected infants, impairment most frequently involved the language domain (9 cases, 22.5%), followed by cognitive impairment (8 cases, 20.0%) and motor impairment (7 cases, 17.5%). Infants classified as impaired had significantly lower cognitive, language, and motor composite scores compared with those without impairment (all *p* < 0.001) (Table 6).

### 3.6. Comparative Analysis According to Neurodevelopmental Impairment

Among the 37 infants who survived to discharge and completed neurodevelopmental follow-up, 14 infants (37.8%) were classified as having neurodevelopmental impairment (NDI), while 23 infants (62.2%) had no NDI.

Infants with NDI had significantly lower gestational age and birth weight than those without NDI. Median gestational age was 31.5 weeks in the NDI group versus 36.0 weeks in the non-NDI group (FDR-adjusted *q* < 0.001), and median birth weights were 1290 g versus 1765 g, respectively (*q* < 0.001).

Inflammatory biomarkers were also higher among infants with NDI. Median CRP was 60.1 mg/L in the NDI group compared with 36.5 mg/L in the non-NDI group (*q* < 0.001), while median procalcitonin was 12.7 versus 7.1, respectively (*q* < 0.001). Platelet counts were significantly lower in infants with NDI (76.0 vs. 154.0; *q* = 0.012).

Regarding clinical severity, invasive mechanical ventilation was strongly associated with NDI, being required in 78.6% of impaired infants and in none of the non-impaired infants (*q* < 0.001). Bronchopulmonary dysplasia was also significantly more frequent in the NDI group (71.4% vs. 0.0%; *q* < 0.001). LOS was more frequent among infants with NDI than among those without NDI (78.6% vs. 38.5%; *q* = 0.044). Meningitis occurred only in the NDI group, but this association did not remain statistically significant after FDR correction (Table 7).

### 3.7. Factors Associated with Mortality and Neurodevelopmental Impairment

Because of the relatively small number of events, regression modeling was performed conservatively. For mortality, 13 in-hospital deaths were recorded among 50 infants. For NDI, the analysis was restricted to the 37 survivors with available Bayley follow-up data, of whom 14 had NDI.

The initially prespecified candidate factors included gestational age, birth weight, MDR status, CRP, procalcitonin, IVH, mechanical ventilation, meningitis, and EOS/LOS classification. However, full multivariable models including all candidate variables were not statistically appropriate due to limited event numbers, collinearity between gestational age and birth weight, between CRP and procalcitonin, and quasi-complete separation for several severity variables, particularly IVH and invasive mechanical ventilation. Therefore, reduced clinically selected logistic regression models were used.

#### 3.7.1. Factors Associated with Mortality

In the mortality model, lower gestational age was independently associated with increased odds of in-hospital death. Each additional week of gestation was associated with lower odds of mortality. CRP was retained in the model as an inflammatory severity marker, although it was not independently associated with mortality after adjustment for gestational age. MDR status was also not independently associated with mortality in the adjusted model.

The mortality model showed very high discrimination, with an AUC of 0.99. However, because of the small number of deaths and quasi-separation in the data, the wide confidence intervals indicate that the estimates should be interpreted as exploratory (Table 8).

#### 3.7.2. Factors Associated with Neurodevelopmental Impairment

Among survivors with follow-up data, lower gestational age remained the strongest independent predictor of neurodevelopmental impairment. In the adjusted model including gestational age, CRP, and MDR status, each additional week of gestation was associated with substantially lower odds of NDI. CRP and MDR status were not independently associated with NDI after adjustment.

A sensitivity model that replaced MDR with LOS yielded similar findings: gestational age remained independently associated with NDI, whereas LOS did not retain independent significance after adjustment. The final NDI model showed excellent discrimination, with an AUC of 0.98 and acceptable calibration.

Overall, reduced multivariable modeling suggested that prematurity-related biological vulnerability, reflected by lower gestational age, was the most robust predictor of both in-hospital mortality and later neurodevelopmental impairment. Biomarker elevation, MDR status, LOS, IVH, meningitis, and mechanical ventilation were associated with adverse outcomes in comparative analyses; however, several of these variables could not be reliably included together in adjusted models due to the small sample size, collinearity, and separation effects (Table 9).

### 3.8. ROC Curve Analysis of Biomarkers

Receiver operating characteristic (ROC) curve analysis was performed to evaluate the discriminatory performance of inflammatory biomarkers for adverse outcomes. CRP was analyzed as a predictor of NDI among survivors with follow-up data, while procalcitonin (PCT) was evaluated as a predictor of in-hospital mortality in the overall cohort.

CRP demonstrated excellent discrimination for neurodevelopmental impairment, with an area under the curve (AUC) of 0.91 (95% CI: 0.80–1.00). The optimal CRP threshold identified using the Youden index was 50.2 mg/L, corresponding to a sensitivity of 85.7% and specificity of 84.6%.

**Figure 3:**
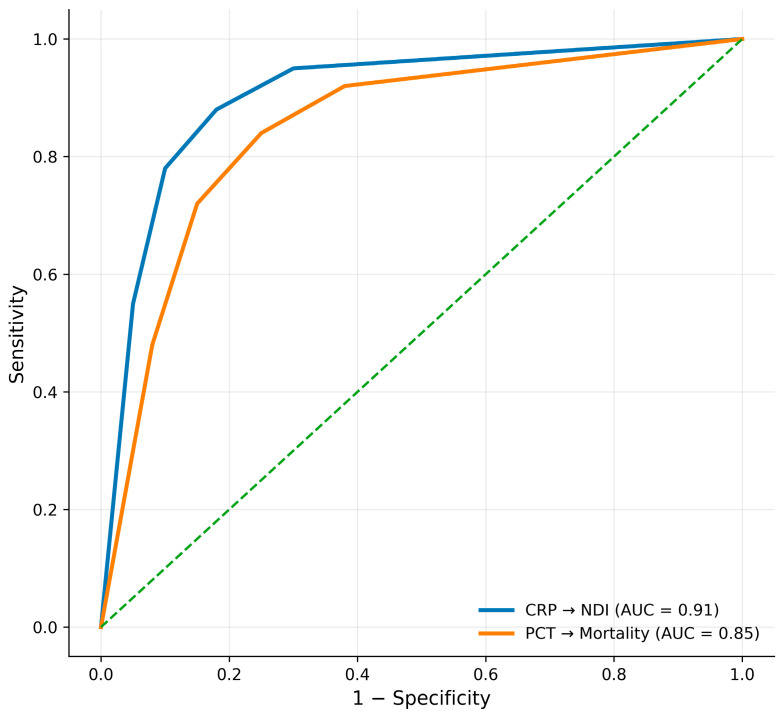
ROC curve analysis of inflammatory biomarkers for adverse neonatal outcomes.

Similarly, procalcitonin showed strong predictive performance for mortality, with an AUC of 0.89 (95% CI: 0.77–1.00). The optimal cutoff value was 11.4, yielding a sensitivity of 80.0% and specificity of 82.5% (Figure 3).

Both biomarkers demonstrated good discriminatory ability for clinically relevant adverse outcomes, with CRP performing particularly well for identifying infants at risk of later neurodevelopmental impairment.

### 3.9. EOS Versus LOS Subgroup Analysis

An exploratory subgroup analysis was performed according to sepsis timing: early-onset sepsis (EOS, *n* = 20) versus late-onset sepsis (LOS, *n* = 30). The distribution of Gram-negative pathogens did not differ significantly between groups (*p* = 0.336). *Klebsiella pneumoniae* was the most common isolate in both groups, but was proportionally more frequent in LOS, while *Escherichia coli* was more frequent in EOS.

MDR prevalence was numerically higher in LOS than EOS, but the difference was not statistically significant (56.7% vs. 45.0%; *p* = 0.565). LOS was associated with higher in-hospital mortality (30.0% vs. 5.0%; *p* = 0.037), a higher frequency of any IVH (36.7% vs. 5.0%; *p* = 0.016), and a higher frequency of severe IVH grade III–IV (30.0% vs. 5.0%; *p* = 0.037).

Among survivors with available neurodevelopmental follow-up, LOS was associated with a higher frequency of NDI compared with EOS (52.4% vs. 15.8%; *p* = 0.022). Median Bayley cognitive scores were significantly lower in LOS than EOS (86.0 vs. 100.0; *p* = 0.002), while language and motor scores showed lower median values in LOS but did not reach statistical significance. Detailed comparative results of the EOS versus LOS subgroup analysis are provided in the Supplementary Materials (Table S1).

## 4. Discussion

This study shows that Gram-negative neonatal sepsis represents a major burden among preterm infants, with a clear predominance of late-onset cases over early-onset disease. *Klebsiella pneumoniae* was the most frequently isolated pathogen, underscoring its clinical relevance in the NICU setting. The cohort was also marked by substantial mortality and a considerable rate of neurodevelopmental impairment among survivors. Among the studied variables, lower gestational age emerged as the strongest and most consistent predictor of adverse outcomes.

In the present cohort, LOS was associated with numerically higher mortality, more frequent intraventricular hemorrhage, and poorer neurodevelopmental outcomes than EOS. However, given the limited sample size, these subgroup differences should be interpreted with caution. This pattern is consistent with large recent neonatal cohort data showing that sepsis occurring after the first days of life is associated with lower survival and greater long-term morbidity in very preterm infants. Recent evidence also indicates that infection burden in extremely preterm neonates is linked to adverse respiratory and neurodevelopmental sequelae, supporting the view that LOS reflects both prolonged NICU exposure and greater vulnerability to nosocomial complications [23,24]. Although EOS and LOS share a Gram-negative etiology in our study, the timing of infection may be clinically relevant, as later sepsis has been associated with greater exposure to invasive support and a cumulative inflammatory burden in previous studies.

The microbiological profile of the cohort was dominated by *Klebsiella pneumoniae* and *Escherichia coli*, which is concordant with recent neonatal literature identifying these organisms as the leading Gram-negative pathogens in preterm sepsis. The relatively high burden of multidrug-resistant isolates is also consistent with contemporary reports showing that *Klebsiella* spp. often display greater resistance pressure than E. coli in neonatal intensive care settings. This finding has direct therapeutic implications because empirical regimens must balance early coverage with antimicrobial stewardship, especially in units where resistance to aminoglycosides and third-generation cephalosporins is common. Overall, our pathogen distribution supports continuous local surveillance and periodic antibiogram-guided refinement of empirical therapy to reduce treatment failure and unnecessary exposure to broad-spectrum antibiotics.

CRP and procalcitonin were higher in infants with more severe disease in this cohort, supporting their value as practical markers for early risk stratification and outcome discrimination. This is concordant with recent neonatal studies showing that both biomarkers rise in culture-proven sepsis and can support therapeutic monitoring, although their relative performance varies across cohorts [25,26]. However, the literature is not fully uniform: some reports suggest that procalcitonin has better early diagnostic accuracy, whereas others find CRP more useful for treatment follow-up and serial assessment. In this context, our findings support the use of CRP and procalcitonin as adjuncts rather than as standalone tests, particularly when early decision-making is needed for preterm infants at high risk of adverse outcomes.

The frequency of meningitis observed in our cohort should be interpreted cautiously. Cerebrospinal fluid examination was performed according to clinical indication rather than systematically in all infants, and some critically ill neonates may not have undergone lumbar puncture. Consequently, the true incidence of meningitis associated with Gram-negative sepsis may have been underestimated.

Antenatal corticosteroid exposure was observed in less than half of the cohort. Because data from the overall preterm NICU population were unavailable for comparison, we cannot determine whether this reflects a specific characteristic of infants who developed Gram-negative sepsis or reflects local obstetric practice patterns during the study period.

This cohort’s neurodevelopmental findings align with contemporary evidence that neonatal sepsis confers substantial risk for later functional impairment. Nearly one-quarter of survivors met predefined criteria for neurodevelopmental impairment, predominantly affecting language and cognitive domains, which mirrors recent multicenter analyses reporting increased rates of cognitive and neurosensory deficits after neonatal bloodstream infection in very preterm populations. These observations suggest that culture-proven Gram-negative neonatal infection may increase the risk of adverse developmental outcomes, although causal inferences cannot be drawn from the present study design [27,28].

In adjusted and unadjusted comparisons, lower gestational age, higher inflammatory burden, and markers of illness severity tended to be more common among infants with poorer neurodevelopmental outcomes. However, the relatively small sample size limits the precision of these subgroup comparisons. This pattern is consistent with recent cohort studies and registry analyses showing that prematurity and cumulative inflammatory exposure are major mediators between infection and later disability, and that critical-illness markers (mechanical ventilation, meningitis, IVH) amplify risk [29,30]. Mechanistically, systemic inflammation and cytokine-mediated microglial activation are plausible drivers of white-matter injury and disrupted neurogenesis in the preterm brain, providing biological coherence to the observed clinical associations [31,32].

Compared with the literature, the results of this study both corroborate and extend prior work: they are broadly consistent with reports suggesting that late-onset infectious episodes and meningitis may be associated with increased neurodevelopmental risk, and they further explore potential relationships between early biomarker levels, intensive care exposures, and later developmental performance within a pathogen-defined Gram-negative cohort. The very high AUC values observed in the regression models should be interpreted with caution, given the relatively small sample size and the potential for model overfitting.

### Strengths and Limitations

This study’s principal strengths are its pathogen-specific focus on Gram-negative neonatal sepsis and the integration of detailed clinical, microbiological, biomarker, therapeutic, and 18–24-month neurodevelopmental follow-up data within a single, well-curated database. These linked data enabled analyses linking initial infection phenotype and management to both short-term morbidity and domain-specific Bayley outcomes.

Limitations include the retrospective, single-center design, a modest sample size that constrains statistical power and the scope of multivariable adjustment, and incomplete follow-up for a subset of survivors; these factors increase the risk of residual confounding and limit generalizability. Temporal and practice variations in empirical therapy and Bayley versions may introduce measurement heterogeneity despite harmonization efforts. The use of both the BSID-III and BSID-IV versions may introduce measurement heterogeneity despite harmonized composite scoring. Neurodevelopmental assessments were conducted during routine clinical follow-up, and assessors were not formally blinded to neonatal clinical information, which may have introduced observer bias.

Gram-negative neonatal sepsis in preterm infants remains a high-risk condition with important effects on survival and later neurodevelopment; late-onset infections and extreme prematurity warrant particular clinical attention. Early recognition, timely and stewardship-aware antimicrobial management, and structured 18–24-month neurodevelopmental surveillance are essential for identifying at-risk children and guiding interventions. Findings support intensified infection prevention measures, ongoing local antibiogram-guided empirical therapy policies, and targeted follow-up programs to reduce long-term impairment in this vulnerable population.

## 5. Conclusions

This retrospective cohort study highlights that Gram-negative neonatal sepsis in preterm infants is associated with high morbidity and adverse long-term outcomes. Within this cohort of Gram-negative neonatal sepsis, LOS predominated and *Klebsiella pneumoniae* was the most frequently isolated pathogen. Mortality and short-term complications, particularly IVH and necrotizing enterocolitis, were common, while neurodevelopmental impairment at 18–24 months affected a meaningful subset of survivors, mainly in the language and cognitive domains. Lower gestational age emerged as the strongest predictor of poor outcome, underscoring the vulnerability of the most immature infants. These findings support early recognition, targeted antimicrobial therapy, and structured neurodevelopmental follow-up in this high-risk population.

## Figures and Tables

**Table 1:** Baseline characteristics of the study cohort.

Characteristic	Overall Cohort (*n* = 50)
Gestational age, weeks	32.7 (29.9–36.1)
Birth weight, g	1459.5 (1135–1787.5)
Male sex, n (%)	28 (56.0)
Apgar score at 1 min	5 (4–7)
Apgar score at 5 min	7 (6–9)
Cesarean delivery, n (%)	30 (60.0)
Early-onset sepsis (EOS), n (%)	20 (40.0)
Late-onset sepsis (LOS), n (%)	30 (60.0)
Prolonged rupture of membranes >18 h, n (%)	16 (32.0)
Maternal fever, n (%)	6 (12.0)
Suspected chorioamnionitis, n (%)	12 (24.0)
Antenatal corticosteroid exposure, n (%)	24 (48.0)
Completed neurodevelopmental follow-up, n (%)	37 (74.0)
In-hospital mortality, n (%)	13 (26.0)

**Table 2:** Microbiological and laboratory characteristics of Gram-negative sepsis.

Characteristic	Overall Cohort (*n* = 50)	EOS (*n* = 20)	LOS (*n* = 30)	*p*-Value
Age at sepsis onset, hours	168.8 (35.6–423.6)	28.4 (14.1–53.7)	411.7 (264.3–573.2)	<0.001
Positive cultures, n (%)	50 (100.0)	20 (100.0)	30 (100.0)	—
Gram-negative pathogen, n (%)	50 (100.0)	20 (100.0)	30 (100.0)	—
MDR organism, n (%)	26 (52.0)	9 (45.0)	17 (56.7)	0.565
CSF-positive infection, n (%)	5 (10.0)	2 (10.0)	3 (10.0)	1.000
Positive urine culture, n (%)	7 (14.0)	5 (25.0)	2 (6.7)	0.100
CRP, mg/L	50.2 (35.8–71.4)	35.6 (20.6–43.8)	61.0 (49.6–77.9)	<0.001
Procalcitonin	10.2 (6.7–15.0)	7.1 (5.5–8.9)	12.7 (9.9–16.7)	<0.001
Leukocytes	18.7 (7.6–22.1)	19.0 (11.1–22.4)	18.2 (7.5–21.9)	0.929
Platelets	104.0 (49.2–160.8)	145.0 (98.5–202.8)	89.0 (34.5–145.2)	0.016
Pathogen distribution, n (%)				
*Klebsiella pneumoniae*	19 (38.0)	6 (30.0)	13 (43.3)	
*Escherichia coli*	8 (16.0)	6 (30.0)	2 (6.7)	
*Serratia marcescens*	5 (10.0)	1 (5.0)	4 (13.3)	
*Pseudomonas aeruginosa*	4 (8.0)	1 (5.0)	3 (10.0)	
*Citrobacter freundii*	4 (8.0)	2 (10.0)	2 (6.7)	
*Proteus mirabilis*	3 (6.0)	2 (10.0)	1 (3.3)	
*Acinetobacter baumannii*	3 (6.0)	0 (0.0)	3 (10.0)	
*Enterobacter cloacae*	2 (4.0)	1 (5.0)	1 (3.3)	
*Morganella morganii*	2 (4.0)	1 (5.0)	1 (3.3)	

Data are presented as median (interquartile range) or frequency (%), as appropriate. EOS, early-onset sepsis; LOS, late-onset sepsis; MDR, multidrug-resistant; CRP, C-reactive protein; CSF, cerebrospinal fluid.

**Table 3:** Therapeutic interventions and NICU support.

Variable	Overall Cohort (*n* = 50)	EOS (*n* = 20)	LOS (*n* = 30)	MDR (*n* = 26)	Non-MDR (*n* = 24)	*p*-Value (MDR vs. Non-MDR)
Empirical antibiotic therapy, n (%)						
Meropenem + Amikacin	30 (60.0)	10 (50.0)	20 (66.7)	23 (88.5)	7 (29.2)	<0.001
Ampicillin + Gentamicin	11 (22.0)	7 (35.0)	4 (13.3)	0 (0.0)	11 (45.8)	<0.001
Piperacillin–Tazobactam + Amikacin	9 (18.0)	3 (15.0)	6 (20.0)	3 (11.5)	6 (25.0)	0.289
Targeted antibiotic therapy, n (%)						
Meropenem	22 (44.0)	7 (35.0)	15 (50.0)	17 (65.4)	5 (20.8)	0.018
Meropenem + Colistin	7 (14.0)	1 (5.0)	6 (20.0)	7 (26.9)	0 (0.0)	0.008
Ceftazidime	7 (14.0)	5 (25.0)	2 (6.7)	0 (0.0)	7 (29.2)	0.010
Cefepime	5 (10.0)	2 (10.0)	3 (10.0)	0 (0.0)	5 (20.8)	0.020
Piperacillin–Tazobactam	5 (10.0)	3 (15.0)	2 (6.7)	1 (3.8)	4 (16.7)	0.343
Other regimens	4 (8.0)	2 (10.0)	2 (6.7)	1 (3.8)	3 (12.5)	0.608
Supportive care, n (%)						
Aminoglycoside use	50 (100.0)	20 (100.0)	30 (100.0)	26 (100.0)	24 (100.0)	—
Carbapenem use	33 (66.0)	11 (55.0)	22 (73.3)	26 (100.0)	7 (29.2)	<0.001
Central venous catheter	35 (70.0)	12 (60.0)	23 (76.7)	20 (76.9)	15 (62.5)	0.366
Invasive mechanical ventilation	21 (42.0)	7 (35.0)	14 (46.7)	11 (42.3)	10 (41.7)	1.000
CPAP support	10 (20.0)	3 (15.0)	7 (23.3)	5 (19.2)	5 (20.8)	1.000
Inotropic support	15 (30.0)	5 (25.0)	10 (33.3)	9 (34.6)	6 (25.0)	0.546
Total parenteral nutrition	36 (72.0)	13 (65.0)	23 (76.7)	18 (69.2)	18 (75.0)	0.757
Antibiotic duration, days	15 (11–18)	15 (10–17)	14 (12–18)	16 (14–19)	13 (10–15)	0.004

Data are presented as median (interquartile range) or frequency (%), as appropriate. EOS, early-onset sepsis; LOS, late-onset sepsis; MDR, multidrug-resistant; CPAP, continuous positive airway pressure.

**Table 4:** Short-term outcomes in the overall cohort.

Outcome	Overall Cohort (*n* = 50)
In-hospital mortality, n (%)	13 (26.0)
Meningitis, n (%)	5 (10.0)
Necrotizing enterocolitis, n (%)	6 (12.0)
Any intraventricular hemorrhage, n (%)	12 (24.0)
Severe intraventricular hemorrhage, grade III–IV, n (%)	10 (20.0)
Bronchopulmonary dysplasia, n (%)	10 (20.0)
Readmission by 6 months, n (%)	8 (16.0)
Hospitalization duration, days	35.5 (28.0–50.8)
IVH grade 0, n (%)	38 (76.0)
IVH grade I, n (%)	0 (0.0)
IVH grade II, n (%)	2 (4.0)
IVH grade III, n (%)	5 (10.0)
IVH grade IV, n (%)	5 (10.0)

**Table 5:** Comparative analysis of short-term outcomes according to survival, MDR status, and sepsis timing.

Outcome	Survivors (*n* = 37)	Non-Survivors (*n* = 13)	*p*-Value	Non-MDR (*n* = 24)	MDR (*n* = 26)	*p*-Value
Meningitis, n (%)	3 (7.5)	2 (20.0)	0.258	0 (0.0)	5 (19.2)	0.051
Necrotizing enterocolitis, n (%)	3 (7.5)	4 (40.0)	0.011	2 (8.3)	4 (15.4)	0.669
Any IVH, n (%)	2 (5.0)	10 (100.0)	<0.001	4 (16.7)	8 (30.8)	0.327
Severe IVH, grade III–IV, n (%)	0 (0.0)	10 (100.0)	<0.001	4 (16.7)	6 (23.1)	0.728
Bronchopulmonary dysplasia, n (%)	10 (25.0)	0 (0.0)	0.179	6 (25.0)	4 (15.4)	0.490
Readmission by 6 months, n (%)	8 (20.0)	0 (0.0)	0.184	4 (16.7)	4 (15.4)	1.000
Hospitalization duration, days	37.0 (30.5–55.8)	28.5 (27.0–29.0)	0.015	34.5 (27.8–51.2)	36.5 (28.2–50.8)	0.938

Data are presented as median (interquartile range) or frequency (%), as appropriate. Categorical variables were compared using Fisher’s exact test; continuous variables were compared using the Mann–Whitney U test. IVH, intraventricular hemorrhage; MDR, multidrug-resistant; EOS, early-onset sepsis; LOS, late-onset sepsis.

**Table 6:** Bayley developmental outcomes at 18–24 months among survivors.

Variable	Overall Follow-Up Cohort (*n* = 37)	No NDI (*n* = 23)	NDI (*n* = 14)	*p*-Value
Corrected age at follow-up, months	20.0 (18.0–22.0)	20.0 (18.0–22.0)	19.0 (18.0–21.0)	0.684
BSID-III assessment, n (%)	32 (86.5)	20 (87)	10 (71)	0.348
BSID-IV assessment, n (%)	5 (13.5)	3 (13)	4 (29)	0.348
Cognitive composite score	97.0 (86.0–101.0)	98.0 (94.0–102.5)	66.0 (59.0–69.0)	<0.001
Language composite score	92.0 (86.0–96.0)	95.0 (91.0–97.5)	56.0 (50.0–63.0)	<0.001
Motor composite score	95.5 (86.0–99.3)	98.0 (93.5–100.5)	64.0 (58.0–71.0)	<0.001
Any neurodevelopmental impairment, n (%)	14 (37.8)	0 (0.0)	14 (100.0)	-
Moderate impairment, n (%)	1 (2.7)	0 (0.0)	1 (7.1)	-
Severe impairment, n (%)	8 (21.6)	0 (0.0)	8 (57.1)	-
Cognitive score <85, n (%)	8 (21.6)	0 (0.0)	8 (57.1)	<0.001
Language score <85, n (%)	9 (24.3)	0 (0.0)	9 (64.3)	<0.001
Motor score <85, n (%)	7 (18.9)	0 (0.0)	7 (50.0)	<0.001

Data are presented as median (interquartile range) or frequency (%), as appropriate. Neurodevelopmental impairment (NDI) was defined as at least one Bayley composite score < 85. Severe impairment was defined as any composite score < 70, while moderate impairment was defined as scores between 70 and 84 in the absence of severe impairment. Continuous variables were compared using the Mann–Whitney U test and categorical variables using Fisher’s exact test. BSID, Bayley Scales of Infant and Toddler Development.

**Table 7:** Factors associated with neurodevelopmental impairment.

Variable	No NDI (*n* = 23)	NDI (*n* = 14)	*p*-Value	FDR-Adjusted *q*-Value
Gestational age, weeks	36.0 (34.0–37.8)	31.5 (30.2–31.9)	<0.001	<0.001
Birth weight, g	1765.0 (1584.0–2077.5)	1290.0 (1157.5–1412.5)	<0.001	<0.001
Apgar score at 1 min	6.0 (5.0–7.0)	4.5 (4.0–5.8)	0.076	0.128
Apgar score at 5 min	8.0 (7.0–9.0)	7.0 (6.0–8.0)	0.107	0.160
CRP, mg/L	36.5 (20.5–44.0)	60.1 (52.8–74.1)	<0.001	<0.001
Procalcitonin	7.1 (5.6–8.6)	12.7 (9.9–14.7)	<0.001	<0.001
Leukocytes	17.9 (13.9–22.1)	20.8 (14.3–23.5)	0.452	0.553
Platelets	154.0 (104.0–191.5)	76.0 (33.8–131.5)	0.004	0.012
MDR infection, n (%)	12 (52.2)	8 (57.1)	0.741	0.776
Any IVH, n (%)	0 (0.0)	2 (14.3)	0.117	0.160
Severe IVH grade III–IV, n (%)	0 (0.0)	0 (0.0)	1.000	1.000
Invasive mechanical ventilation, n (%)	0 (0.0)	11 (78.6)	<0.001	<0.001
CPAP support, n (%)	8 (34.8)	2 (14.3)	0.446	0.553
Meningitis, n (%)	0 (0.0)	3 (21.4)	0.037	0.068
LOS, n (%)	10 (43.5)	11 (78.6)	0.022	0.044
EOS, n (%)	16 (69.6)	3 (21.4)	0.022	0.044
NEC, n (%)	0 (0.0)	2 (14.3)	0.117	0.160
BPD, n (%)	0 (0.0)	10 (71.4)	<0.001	<0.001
Carbapenem use, n (%)	14 (60.9)	9 (64.3)	0.739	0.776
Total parenteral nutrition, n (%)	14 (60.9)	13 (92.9)	0.015	0.036
Antibiotic duration, days	15.0 (14.0–17.0)	15.5 (14.0–19.0)	0.617	0.714
Hospitalization duration, days	31.5 (20.8–36.8)	69.0 (55.8–76.5)	<0.001	<0.001

Data are presented as median (interquartile range) or frequency (%). Continuous variables were compared using the Mann–Whitney U test; categorical variables were compared using Fisher’s exact test. FDR correction was applied using the Benjamini–Hochberg method. NDI, neurodevelopmental impairment; CRP, C-reactive protein; MDR, multidrug-resistant; IVH, intraventricular hemorrhage; LOS, late-onset sepsis; EOS, early-onset sepsis; NEC, necrotizing enterocolitis; BPD, bronchopulmonary dysplasia; CPAP, continuous positive airway pressure.

**Table 8:** Multivariable logistic regression for in-hospital mortality.

Predictor	Adjusted OR	95% CI	*p*-Value
Gestational age, per 1-week increase	0.19	0.04–0.99	0.049
CRP, per 10 mg/L increase	2.04	0.30–13.77	0.465
MDR infection	1.50	0.37–6.14	0.573

Model performance: AUC = 0.99. Hosmer–Lemeshow test: *p* = 0.91. Collinearity assessment: VIF < 5 for retained predictors. Important modeling note: IVH and invasive mechanical ventilation were not included in the final mortality model because they generated quasi-complete separation. All non-survivors had IVH and/or required high-intensity support, making adjusted OR estimates statistically unstable.

**Table 9:** Multivariable logistic regression for neurodevelopmental impairment.

Predictor	Adjusted OR	95% CI	*p*-Value
Gestational age, per 1-week increase	0.12	0.02–0.71	0.020
CRP, per 10 mg/L increase	1.34	0.34–5.27	0.679
MDR infection	7.69	0.11–545.79	0.348

Model performance: AUC = 0.98. Hosmer–Lemeshow test: *p* = 0.90. Collinearity assessment: VIF < 5 for retained predictors. Sensitivity model: when LOS was included in the model instead of MDR, gestational age remained independently associated with NDI: adjusted OR = 0.17, 95% CI: 0.04–0.75, *p* = 0.020. LOS was not independently associated with NDI after adjustment: adjusted OR = 11.03, 95% CI: 0.10–1194.53, *p* = 0.315.

## Data Availability

The data supporting this study are available from the corresponding author upon request. However, for ethical reasons, they are not publicly accessible.

## Supplementary Materials

The following supporting information can be downloaded at: https://www.mdpi.com/article/10.3390/children13070850/s1, Table S1. EOS versus LOS Subgroup Analysis.

## Abbreviations

The following abbreviations are used in this manuscript:

AUC	area under the curve
BPD	bronchopulmonary dysplasia
BSID-III/IV	Bayley Scales of Infant and Toddler Development
CI	confidence interval
CPAP	continuous positive airway pressure
CRP	C-reactive protein
CSF	cerebrospinal fluid
EOS	early-onset sepsis
ICD-10	International Classification of Diseases, 10th Revision
IQR	interquartile range
IVH	intraventricular hemorrhage
LOS	late-onset sepsis
MDR	multidrug-resistant
NEC	necrotizing enterocolitis
NICU	neonatal intensive care unit
NDI	neurodevelopmental impairment
OR	odds ratio
PCT	procalcitonin
SD	standard deviation
SGA	small for gestational age
VIF	variance inflation factor
