# Supplementary material for: From Gram-Negative Neonatal Sepsis to Neurodevelopmental Impairment: A Retrospective Cohort Study in Preterm Infants
Source: Children (Basel). 2026 Jun 24;13(7):850. doi: 10.3390/children13070850 (PMC13406810; doi:10.3390/children13070850)
Supplement: Supplementary file 1 [file children-13-00850-s001.zip › children-4366636-supplementary.pdf]

Supplementary Material

Table S1. EOS versus LOS Subgroup Analysis

Variable	EOS (n = 20)	LOS (n = 30)	*p*-value
MDR infection, n (%)	9 (45.0)	17 (56.7)	0.565
In-hospital mortality, n (%)	1 (5.0)	9 (30.0)	0.037
Any IVH, n (%)	1 (5.0)	11 (36.7)	0.016
Severe IVH grade III–IV, n (%)	1 (5.0)	9 (30.0)	0.037
Meningitis, n (%)	2 (10.0)	3 (10.0)	1.000
Hospitalization duration, days	35.5 (18.8–39.5)	35.0 (29.0–54.0)	0.184
CRP, mg/L	35.6 (20.6–43.8)	61.0 (49.6–77.9)	<0.001
Procalcitonin	7.1 (5.5–8.9)	12.7 (9.9–16.7)	<0.001
NDI among survivors with follow-up, n/N (%)	3/19 (15.8)	11/21 (52.4)	0.022
Bayley cognitive score	100.0 (95.5–103.0)	86.0 (69.0–97.0)	0.002
Bayley language score	95.0 (90.0–96.5)	86.0 (63.0–95.0)	0.082
Bayley motor score	98.0 (93.0–100.0)	86.0 (71.0–97.0)	0.069

Pathogen distribution

Pathogen	EOS (n = 20)	LOS (n = 30)
Klebsiella pneumoniae	6 (30.0)	13 (43.3)
Escherichia coli	6 (30.0)	2 (6.7)
Serratia marcescens	1 (5.0)	4 (13.3)
Pseudomonas aeruginosa	1 (5.0)	3 (10.0)
Citrobacter freundii	2 (10.0)	2 (6.7)
Proteus mirabilis	2 (10.0)	1 (3.3)
Acinetobacter baumannii	0 (0.0)	3 (10.0)
Enterobacter cloacae	1 (5.0)	1 (3.3)
Morganella morganii	1 (5.0)	1 (3.3)
Pathogen	EOS (n = 20)	LOS (n = 30)

Pathogen distribution comparison: p = 0.336.

Data are presented as median (interquartile range) or frequency (%). Continuous variables were compared using the Mann–Whitney U test; categorical variables were compared using Fisher's exact test, except pathogen distribution, assessed using χ^2 test. EOS, early-onset sepsis; LOS, late-onset sepsis; MDR, multidrug-resistant; IVH, intraventricular hemorrhage; CRP, C-reactive protein; NDI, neurodevelopmental impairment.
